# Pharmacokinetics, Safety and Pharmacokinetics/Pharmacodynamics Analysis of Omadacycline in Chinese Healthy Subjects

**DOI:** 10.3389/fphar.2022.869237

**Published:** 2022-04-21

**Authors:** Haijing Yang, Zhiwei Huang, Yuancheng Chen, Yusong Zhu, Guoying Cao, Jingjing Wang, Yan Guo, Jicheng Yu, Jufang Wu, Lichuan Liu, Jun Deng, Jing Liu, Harald Reinhart, Jing Zhang, Xiaojie Wu

**Affiliations:** ^1^ Phase I Clinical Research Center, Huashan Hospital, Fudan University, Shanghai, China; ^2^ Key Laboratory of Clinical Pharmacology of Antibiotics, National Health Commission, Shanghai, China; ^3^ National Clinical Research Center for Geriatric Diseases (Huashan Hospital), Shanghai, China; ^4^ Institute of Antibiotics, Huashan Hospital, Fudan University, Shanghai, China; ^5^ Zai Lab (Shanghai) Co., Ltd., Shanghai, China

**Keywords:** double-peak absorption, compartment model, Monte Carlo simulation, methicillin-resistant *Staphylococcus aureus*, acute bacterial skin and skin structure infections, community-acquired bacterial pneumonia, omadacycline

## Abstract

**Objective:** Omadacycline is a new type of aminomethylcycline antibiotic, having a broad antibacterial spectrum. But the pharmacokinetic characteristics and safety profile of the Chinese population remain unknown. It is also unclear whether the US-approved treatment regimen is applicable for the Chinese population.

**Methods:** In a randomized, double-blinded, placebo-controlled dose-escalated trial, the pharmacokinetics of omadacycline was evaluated by a non-compartmental and compartmental model. Monte Carlo simulations were performed using the pharmacokinetic data from the Chinese population to evaluate the probability of target attainment (PTA) and the cumulative fraction of response (CFR) of the US FDA-approved dose regimen.

**Results:** The three-compartment model successfully described the rapid distribution and slow elimination of omadacycline after the intravenous infusion (i.v.). The double-peak concentration-time curve of the oral absorption (p.o.) was explained by the two-compartment model with two absorption compartments. The steady-state AUC of 100 mg omadacycline i.v. and 300 mg omadacycline p. o. were 12.1 and 19.4 mg h/L, respectively. Pharmacokinetics/pharmacodynamics (PK/PD) analysis showed that the omadacycline dosing regimen with a loading dose (200 mg i.v. q24 h, 100 mg i.v. q12 h, 450 mg p. o. q24 h × 2 days or 300 mg p. o. q12 h) and maintenance dose (100 mg i.v. q24 h or 300 mg p. o. q24 h) could cover the main pathogens of the indications acute bacterial skin and skin structure infections (ABSSSI) and community-acquired bacterial pneumonia (CABP): *Staphylococcus aureus* and *Streptococcus pneumoniae*. Also, omadacycline had showed a good safety profile in the Chinese population.

**Conclusions:** With the evidence provided, omadacycline could be a novel treatment option to Chinese patients with ABSSSI and CABP.

## Introduction

Omadacycline is a new type of aminomethylcycline antibiotic, a derivative of minocycline, and with intravenous and oral formulations. Similar to other tetracycline drugs, omadacycline inhibits bacterial protein synthesis by binding to the 30S ribosomal subunit. It has a broad antibacterial spectrum, including Gram-positive bacteria, Gram-negative bacteria, anaerobic bacteria, and atypical pathogens, and can overcome the most common mechanisms of tetracycline resistance (such as efflux pump: *TetK* and ribosome protective protein: *TetM*) (Draper et al., 2014; Karlowsky et al., 2019; Pfaller et al., 2020). Omadacycline was first approved by the US FDA in 2018 for the treatment of adult patients with acute bacterial skin and skin structure infections (ABSSSI) and community-acquired bacterial pneumonia (CABP) and was recently approved by the Center for Drug Evaluation of China.

The oral bioavailability of omadacycline is 34.5% (Sun et al., 2016) and food, especially high-fat meals and dairy products, will reduce its absorption (Tzanis et al., 2017). Omadacycline has a wide tissue distribution, and the volume of distribution after intravenous administration is about 200 L (Gotfried et al., 2017; Berg et al., 2018; Kovacs et al., 2020a; Kovacs et al., 2020b). It also has higher concentrations in the bone, liver, skin, and lungs (Lin et al., 2017). The terminal half-life of omadacycline is 11.3–17.1 h (Berg et al., 2018; Kovacs et al., 2020b), with extremely low liver metabolism. In terms of intravenous injection of omadacycline, 27% is excreted from urine as a prototype (Berg et al., 2018). Omadacycline has been described in a three-compartment model in the population pharmacokinetic analysis with gender as a significant covariate for clearance (Lakota et al., 2020). *In vitro* and animal studies have reflected that the best PK/PD index is the area under the concentration-time curve/minimum inhibitory concentration (AUC/MIC) (Lepak et al., 2017; Lepak et al., 2020; Noel et al., 2021).

Although pharmacokinetic studies of omadacycline have been carried out in the western population, the pharmacokinetic characteristics and safety of omadacycline in the Chinese population are still unclear. It is reported that omadacycline exhibited very good activity against Chinese isolates, suggesting that omadacycline could be an option for the treatment of skin infections and pneumonia in Chinese patients (Carvalhaes et al., 2019; Dong et al., 2020). This study evaluated the safety and pharmacokinetic characteristics of omadacycline following intravenous and oral administrations at single and multiple doses in healthy Chinese subjects and evaluated whether the US FDA-approved dose regimen is suitable for the Chinese population from PK/PD perspective.

## Materials and Methods

**Subjects** The study protocol and the informed consent form was reviewed and approved by the Ethics Committee of Huashan Hospital Fudan University. The trial was carried out in strict accordance with the GCP, the *Declaration of Helsinki*, and applicable laws and regulations (clinical trial registration number: CTR20190089). Prior to any procedures, the study physician has fully informed the subjects of the study protocol and possible safety issues, and all subjects have signed the informed consent form.

Inclusion criteria: healthy male (weight ≥50 kg) or female (weight ≥45 kg) subjects aged 18 to 45, with a body mass index (BMI) within the range of 18–26 kg/m^2^. The main exclusion criteria included: history of any allergies including tetracycline drugs; gastrointestinal surgery that may affect the absorption of the study drug or gastrointestinal diseases within 3 months; clinical significant abnormal results in physical examination, vital signs, 12-lead electrocardiogram (ECG), or laboratory tests (including hematology, chemistry, urinalysis, and pregnancy test), etc.

**FIGURE 1:**
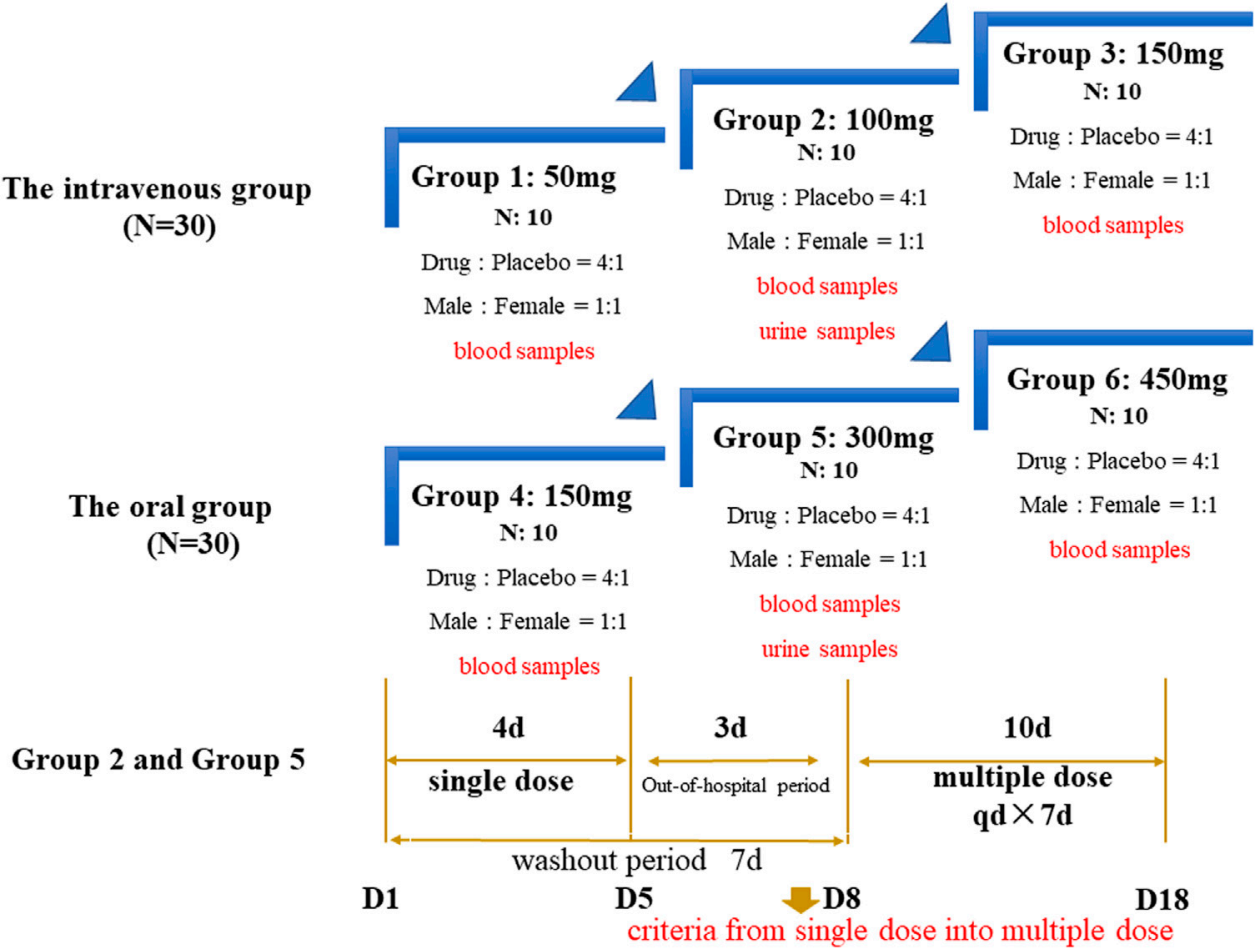
Dose regimen of the phase I omadacycline trial.

**Study Design** This study was a single-center, randomized, double-blinded placebo-controlled, dose-escalation phase I clinical study. As shown in Figure 1A total of 60 healthy subjects were planned to be enrolled in this study. They were separated into the intravenous group and oral group: each included three groups, with ten subjects (male: female = 1:1) in each group, using a dose-escalation design between the groups. If two or more subjects in the same group experienced the same category of drug-related grade 3 or above adverse events (AE), or four or more subjects in the same group experienced the same category of drug-related Grade 2 AEs, the next dose would not be performed and the study would be suspended. If the halting criteria were not met, the next dose would be continued.

For intravenous 100 mg group and oral 300 mg group, both had single dose and multiple dose administration, and the washout period was 7 days. After the subjects were assessed and confirmed to meet the criteria from single dose into multiple dose, they could directly enter into multi-dose administration. These two doses were considered clinical maintenance doses, so urine samples were also collected besides blood samples after a single dose. The subjects in the intravenous infusion group could have a standardized breakfast before administration, while the subjects in the oral group were fasted for at least 10 h before taking the drug, and dry fasted for 2 h after drug administration. Omadacycline lyophilized powder injection (100 mg/bottle, batch number: 3181102) and tablets (150 mg/tablet, batch number: B135A21801D), placebo lyophilized powder injection (batch number: 320181108) and tablets (batch number: B151A11801D) were provided by Zai Lab (Shanghai) Co., Ltd.

**Sample Collection and Testing** Subjects in the intravenous administration group were sampled at 19 (single dose) to 34 (multiple doses) time points, i.e., pre-dose (within 60 min), 15 min, 33 min (immediately after the completion of intravenous infusion), 45 min, 1, 1.25 h (single dose administration group only), 1.5, 1.75, 2, 4, 6, 8, 12, 18, 24, 36, 48, 72, and 96 h after the start of intravenous infusion. Subjects on oral administration group were sampled at 16 (single dose) to 30 (multiple doses) time points: pre-dose, 30 min, 1, 1.5, 2, 3, 4, 6, 8, 12, 18, 24, 36, 48, 72, and 96 h after administration. In the multiple dose group, blood samples were collected 24 h after administration on day 1, and trough blood samples were collected 2 days before the last dose. When the 100 mg intravenous group and 300 mg oral group were administered at a single dose, urine samples were collected before dosing (-1–0 h) and at 0–4, 4–8, 8–12, 12–24 and, 24–48 h after dosing. The concentrations of omadacycline in plasma and urine were quantified using validated UPLC-MS/MS methods using CSH C18 (50 × 2.1 mm, 1.7 μm). Gradient elution was performed to separate drugs using 30 mM ammonium formate aqueous solution and methanol-acetonitrile solution (v/v, 1:1) as the mobile phase A and B with a flow rate of 0.6 ml/min. The linear range was 0.0200–2.00 mg/L for plasma and 0.0800–20.0 mg/L for urine. Samples below the quantification limit (BQL) were excluded from the analysis.

**Pharmacokinetics Analysis** Pharmacokinetics (PK) parameters of omadacycline were calculated with Phoenix WinNonlin software (version 8.0, Certara Co. Ltd., United States). PK parameters of the non-compartmental model included peak concentration or steady-state peak concentration (C_max_/C_ss, max_), time to peak or steady-state time to peak (T_max_/T_ss, max_), and area under the concentration-time curve from 0 to 24 (AUC_0-24_), the AUC from 0 to infinity (AUC_0-inf_), the steady-state dosing interval AUC (AUC_tau_), the clearance or the steady-state clearance (CL/CL_ss_ for intravenous infusion; CL/F or CL_ss_/F for oral administration, F was bioavailability), the volume of distribution or steady-state volume of distribution (V_z_/V_ss_ for intravenous infusion; V_z_/F or V_ss_/F for oral administration), elimination half-life (t_1/2_), cumulative excretion fraction (*fe*), renal clearance (CL_R_) and accumulation ratio (R_ac_). The PK parameter AUC_0-inf_ of single dose oral and intravenous administration was normalized by dose levels, and the result of dose normalization was used to calculate the absolute bioavailability (oral 150 mg group *vs* intravenous 50 mg group, oral 300 mg group *vs* intravenous 100 mg group, and oral 450 mg group *vs* intravenous 150 mg group).

Intravenous infusion adopted a three-compartment model. PK parameters included: central compartment volume of distribution (V_1_), peripheral compartment volume of distribution (V_2_, V_3_), central compartment clearance (CL), and peripheral compartment clearance (CL_2_, CL_3_). The oral administration adopted a two-compartment model with two first-order absorption compartments were used to describe the double-peak curve. k_a1_ and k_a2_ were the rates of omadacycline from the absorption compartments to the central compartment, P_1_ was the ratio of the drug amount in the first absorption compartment, and T_lag_ was the lagging absorption time of the second absorption compartment.

**Linear Relationship Evaluation** Whether the increase in the value of AUC_0-inf_ presented a linear relationship with the increase in the dose of omadacycline was investigated with the power model (Eq. 1).

Formula 1 :ln(AUC0-inf) = ln(α)+β×ln(Dose) (1)

When β 95% confidence interval contained 1, it was considered that AUC_0-inf_ had a linear relationship with dose.

**Pharmacokinetic Simulation** PK parameters of the three-compartment model established by intravenous administration of 50–150 mg and Phoenix WinNonlin software “PK Model” module were used to extrapolate and simulate the AUC_0-24_ of a single dose intravenous infusion of 200 mg omadacycline (1 h infusion) in healthy Chinese subjects. The Phoenix WinNonlin software “NonParametric Superposition” module was used to simulate the AUC_0-24_ of two doses of omadacycline intravenously infused at 100 mg (0.5 h infusion, q12 h) and orally administered at 300 mg (q12 h) (Lin et al., 2019; Huang et al., 2021).

**Monte Carlo Simulation** R software (version 4.0.4) was used to simulate the PK parameter AUC_0-24_ 1000 times according to the normal distribution, and to calculate the probability of target attainment (PTA) and cumulative fraction of response (CFR) of the US FDA-approved dose regimen (loading dose: 200 mg i.v. q24 h, 100 mg i.v. q12 h, 450 mg p. o. q24 h or 300 mg p. o. q12h; maintenance dose: 100 mg i.v. q24 h or 300 mg p. o. q24 h). For ABSSSI, free AUC_0-24_/MIC (*f* AUC_0-24_/MIC) was used for PK/PD analysis. *f* AUC_0-24_/total AUC_0-24_ = 0.79 (Lin et al., 2017) and PK/PD target of *f* AUC_0-24_ = 12.5 (96% early clinical response) was derived from patients with ABSSSI (unpublished data). For CABP, the epithelial lining fluid (ELF) could better assess the exposure-response relationship of antibiotics in lower respiratory tract infections. ELF AUC_0-24_/MIC is used for PK/PD analysis. ELF AUC_0-24_/plasma AUC_0-24_ = 1.47 (Gotfried et al., 2017). Median ELF AUC_0-24_/MIC (1-log_10_ kill) from mouse pneumonia model for methicillin-resistant *Staphylococcus aureus* (MRSA) and methicillin-sensitive *Staphylococcus aureus* (MSSA) infection were 6.30 and 1.86 (Lepak et al., 2020), and 15.46 for *Streptococcus pneumoniae* infection (Lepak et al., 2017) and 8.91 for *Haemophilus influenzae* infection in an *in vitro* model. The distribution frequency of MIC was provided by the microbiology group of the Institute of Antibiotics, Huashan Hospital Fudan University, including *Staphylococcus aureus* (98 strains of MRSA and 102 strains of MSSA), *Streptococcus pneumoniae* (103 strains, of which 68 strains were resistant to penicillin and 96 strains were resistant to macrolides) and *Haemophilus influenzae* (101 strains, 50 β-lactamase positive strains) (Lepak et al., 2017).

**Safety Evaluation** Throughout the study, the subjects remained in the hospital for safety evaluations except for the 3-day out-of-hospital washout period. AEs, vital signs, physical examinations, laboratory tests (including hematology, chemistry, and urinalysis), and 12-lead ECG were monitored. The severity of adverse events was assessed according to the Common Terminology Criteria for Adverse Events (CTCAE) (V 4.03) (U.S. Department of Health and Human Services, 2010).

## Results

**Subject** From 14 January 2019 to 30 May 2019, a total of 297 subjects were screened and 63 subjects were enrolled, of which 61 subjects completed the study, 11 subjects entered the placebo group, and two subjects withdrew from the study early in the 450 mg oral group for vomiting within 1 h after the administration. The male and female ratios of the enrolled subjects were balanced. The average age of the subjects in each group ranged from 24.1 (±3.7) to 33.0 (±8.5) years, the average weight ranged from 59.3 (±8.9) to 72.7 (±3.7) kg, and the average BMI ranged from 21.7 (±2.3) to 23.9 (±0.57) kg/m^2^. The demographics of the subjects were similar among the groups (Supplementary Table S1).

**FIGURE 2:**
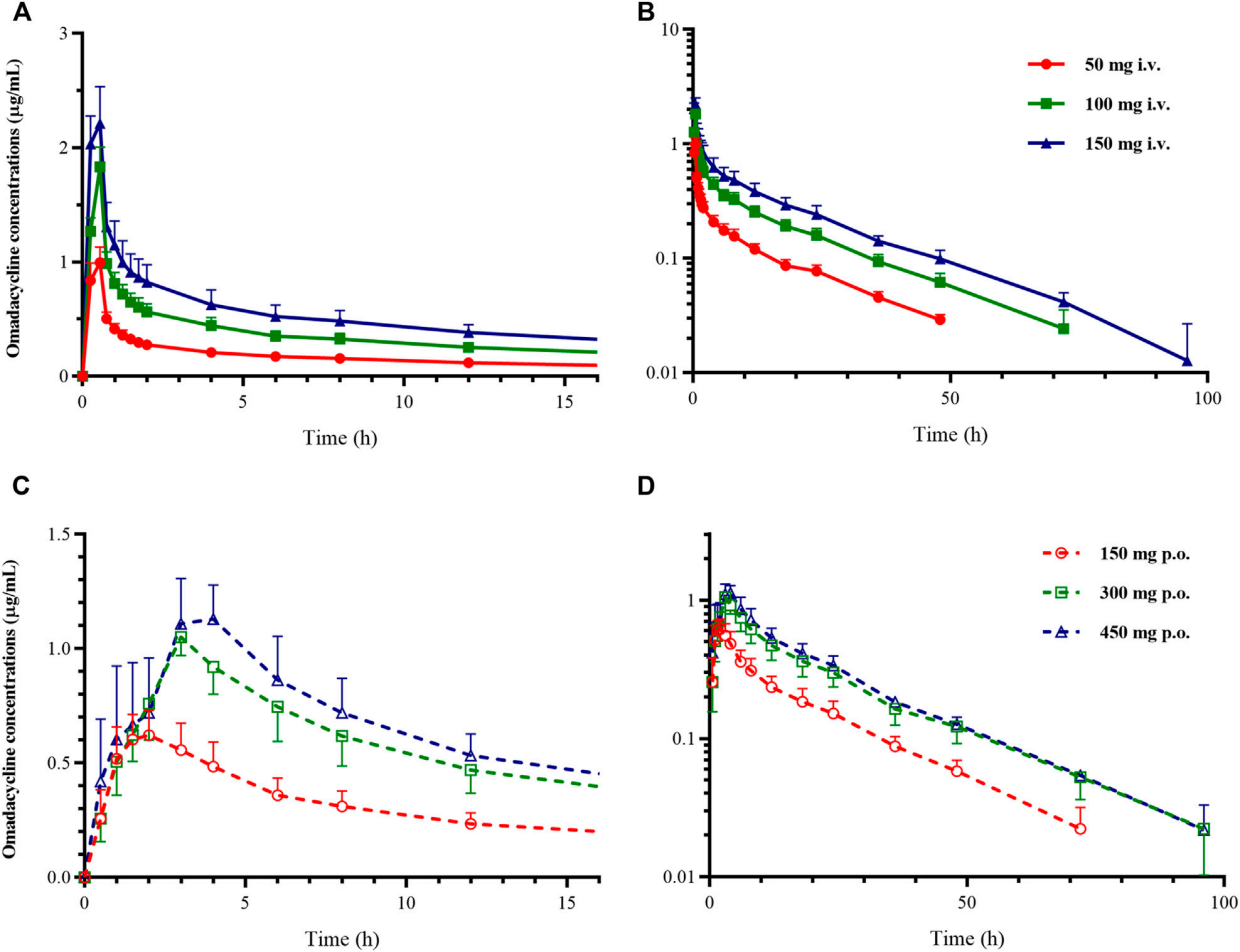
Mean plasma concentration-time curve of omadacycline. **(A)** Constant coordinate graph of intravenous administration of omadacycline (50, 100, and 150 mg); **(B)** semi-logarithmic graph of intravenous administration of omadacycline (50, 100, and 150 mg); **(C)** constant coordinate graph of oral administration of omadacycline (150, 300, and 450 mg); and **(D)** semi-logarithmic graph of oral administration of omadacycline (150, 300, and 450 mg).

**Pharmacokinetic Analysis** In this study, the PK analysis included 1288 plasma concentrations from 46 subjects. Two subjects each from 300 to 450 mg oral group were excluded from the PK analysis for vomiting within 1 h after administration. As shown in Figure 2 for the concentration-time curve of omadacycline, the intravenous administration of omadacycline (50, 100, and 150 mg) reached the peak at the end of the infusion (Figure 1A), and then rapidly distributed. The 150 mg, 300 mg, and 450 mg oral administration groups peaked at 1.75, 3, and 4 h, respectively (Figure 2A,C); all the groups were eliminated slowly at the terminal phase (Figure 2B,D). The t_1/2_ of the six groups were relatively close, ranging from 17.2 to 20.7 h (Table 1), and the t_1/2_ for multi-dose administration was slightly longer, at 25.5 and 25.6 h. CL (8.50–8.83 L/h) and V_z_ (220–248 L) were similar in the intravenous groups, while CL/F (increased from 14.5 L/h to 20.1 L/h) and V_z_/F (increased from 396 to 573 L) increased with the doses in the oral groups, which may be attributed to the linear relationship between AUC_0-inf_ and dose in the intravenous group (β = 1.04, 95% confidence interval [0.95, 1.14]), and the non-linear relationship between AUC_0-inf_ and dose in the oral group (β = 0.73, 90% Cmax 1.8–1.9 mg/L [0.58, 0.88]). The renal excretion rate of the 100 mg intravenous group was 31.6%, while of the 300 mg oral group was 18.2%, but the renal clearance rates of the two groups were similar (3.1 *vs* 3.3 L/h). The R_ac_ of the oral administration group was higher than that of the intravenous administration group (R_ac(AUC)_: 1.83 *vs* 1.51; R_ac(Cmax)_: 1.49 *vs* 1.07). The absolute bioavailability was 62.3, 57.2, and 42.0%, respectively, in the 150 mg, 300 mg, and 450 mg oral administration group.

**TABLE 1:** Pharmacokinetic parameters of omadacycline following intravenous and oral administrations by the non-compartmental model.

		Intravenous Administration				Oral Administration^a^ (mg)			
		50 mg	100 mg		150 mg	150 mg	300 mg		450 mg
			Single dose	Steady state^b^			Single dose	Steady state^b^	
*n*		8	8	8	8	8	6	8	6
C_max_ (μg/ml)		1.01 (14.9)	1.83 (9.5)	1.99 (8.4)	2.23 (13.8)	0.644 (16.6)	1.05 (7.6)	1.33 (15.4)	1.21 (11.8)
T_max_ (h)		0.55 (0.25, 0.57	0.61 (0.57, 0.65)	0.54 (0.25,0.55)	0.55 (0.25, 0.57)	1.75 (1.00, 3.00)	3.00 (3.00, 3.00	3.00 (1.50,4.00)	3.98 (2.98, 3.98)
AUC (μg•h/mL)	AUC_0-24_	3.82 (10.4)	7.79 (11.4)	12.1 (16.5)	11.4 (17.1)	6.80 (19.7)	12.4 (16.9)	19.4 (20.3)	14.2 (15.6)
	AUC_0-inf_	5.70 (8.1)	11.9 (13.1)	\	18.0 (15.6)	10.7 (17.7)	20.4 (19.2)	\	22.7 (12.5)
t_1/2_ (h)		17.2 (11.8)	19.1 (10.9)	25.5 (14.5)	20.1 (14.3)	18.9 (16.8)	20.7 (7.0)	25.6 (7.6)	19.7 (11.5)
CL (L/h)		8.83 (8.6)	8.51 (12.6)	8.47 (16.1)	8.50 (14.3)	14.5 (16.5)	15.2 (22.3)	16.1 (22.1)	20.1 (11.8)
C_ss,min_ (μg/ml)		\	\	0.276 (16.7)	\	\	\	0.487 (21.1)	\
V_z_ (L)		220 (17.8)	233 (15.0)	238 (13.4)	248 (22.4)	396 (23.6)	454 (23.7)	537 (19.3)	573 (19.4)
R_ac (AUC)_		\	\	1.51 (3.6)	\	\	\	1.83 (14.8)	\
R_ac (Cmax)_		\	\	1.07 (9.8)	\	\	\	1.49 (12.1)	\
*F*e(%)		\	31.6 (15.7)	\	\	\	18.2 (16.7)	\	\
CL_R_ (L/h)		\	3.13 (18.4)	\	\	\	3.32 (18.3)	\	\

a The corresponding PK, parameters for oral administration are CL/F, V_z_/F, and F was bioavailability;

b The corresponding PK, parameters for steady state are C_ss,max_, T_ss,max_, AUC_0-tau_, CL_ss_, or CL_ss_/F, V_ss_ or V_ss_/F; T_max,_ column is displayed as Median (Minand Max), and the other columns are displayed as mean (CV%). AUC_0-24_, area under the concentration-time curve from 0 to 24; AUC_0-inf_, the AUC from 0 to infinity; AUC_tau_, the steady-state dosing interval AUC; C_max_ or C_ss, max_, peak concentration or steady-state peak concentration; C_ss,min_, steady-state trough concentration; CL or CL_ss_, the clearance or the steady-state clearance; CL_R_, renal clearance; *fe*, cumulative excretion fraction; R_ac_, accumulation ratio; t_1/2_, elimination half-life; T_max_ or T_ss, max_, time to peak or steady-state time to peak; V_z_ or V_ss_, volume of distribution or steady-state volume of distribution.

**FIGURE 3:**
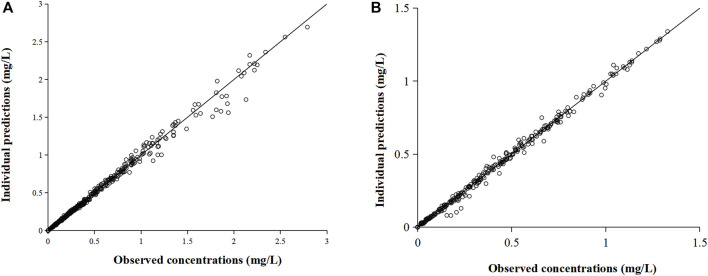
Comparison of individual predicted value and observed value of omadacycline in the compartmental model. **(A)** Intravenous administration and **(B)** oral administration.

The three-compartment model (Supplementary Figure S1A) well described the PK characteristics of the rapid distribution of omadacycline after intravenous administration and the biexponential elimination. The two-compartment model with two absorption compartments (Supplementary Figure S1B) well described the double-peak concentration-time curve of omadacycline. The comparison between the individual predicted value and the observed value was shown in Figure 3, and the PK parameters of the compartmental model were shown in Table 2. Intravenous infusion of omadacycline showed a larger V_3_ (109–134 L), indicating that it may be widely distributed in various tissues in the human body. At the same time, it showed smaller CL_3_ (14.7–22.0 L/h, transport rate k_31_ from the peripheral compartment V_3_ to the central compartment V_1_ was CL_3_/V_3_), indicating that omadacycline that may be distributed in deep tissues and is slowly released into the blood. The two-absorption compartments model could well describe the slow and rapid absorption of oral omadacycline in the 300 mg group and the double-peak absorption in the 450 mg group (Supplementary Figure S2 for comparison between some individual predicted values and observed values), but the absorption rate varied greatly among individuals, with CV% exceeding 100%.

**TABLE 2:** Pharmacokinetic parameters of omadacycline following intravenous and oral administrations by the compartmental model.

	Intravenous Administration			Oral administration^a^		
	50 mg	100 mg	150 mg	150 mg	300 mg	450 mg
***n***	8	8	8	8	6	6
**V**_**1**_ **(L)**	17.2 (24.3)	19.7 (24.3)	19.3 (14.9)	110 (54.6)	103 (17.0)	146 (28.2)
**V**_**2**_ **(L)**	58.1 (23.4)	63.2 (6.8)	71.7 (21.0)	215 (28.4)	244 (30.1)	281 (24.2)
**V**_**3**_ **(L)**	115 (29.2)	109 (23.5)	118 (31.7)	\	\	\
**CL (L/h)**	8.76 (8.8)	8.5 (12.4)	8.48 (14.2)	14.5 (17.3)	15.2 (20.7)	20.2 (11.7)
**CL**_**2**_ **(L/h)**	65.6 (17.0)	78.9 (9.1)	99.8 (16.4)	38.8 (39.7)	34.2 (23.8)	41.5 (38.2)
**CL**_**3**_ **(L/h)**	18.7 (29.2)	18.7 (23.1)	20.8 (43.8)	\	\	\
**k**_**a1**_ **(h** ^**−1**^ **)**	\	\	\	0.967 (63.8)	0.342 (57.2)	1.09 (98.5)
**k**_**a2**_ **(h** ^**−1**^ **)**	\	\	\	1.95 (117.8)	4.58 (72.1)	2.29 (123.8)
**P**_**1**_	\	\	\	0.581 (35.8)	0.786 (10.8)	0.657 (32.5)
**T**_**lag**_ **(h)**	\	\	\	0.678 (54.2)	1.85 (15.7)	2.19 (14.6)

a V_1_, V_2_, CL_1,_ and CL_2_ for oral administration are V_1_/F, V_2_/F, CL_1_/F, and CL_2_/F, respectively; the PK parameter is represented as mean (CV%); CL, central compartment clearance; CL_2_ and CL_3,_ peripheral compartment clearance; k_a1_ and k_a2,_ the rates of omadacycline from the absorption compartments to the central compartment; P_1,_ the ratio of the drug amount in the first absorption compartment; T_lag,_ the lagging absorption time of the second absorption compartment; V_1,_ central compartment volume of distribution; V_2_ and V_3,_ peripheral compartment volume of distribution.

**Pharmacokinetic Simulation** The AUC_0-24_ of omadacycline intravenously infused at 200 mg q24 h (1 h infusion) was 15.3 ± 1.9 mg h/L (*n* = 24), which was derived from the extrapolation and simulation with the compartment model parameters. The AUC_0-24_ of omadacycline intravenously infused at 100 mg q12 h (0.5 h infusion) was 13.2 ± 1.5 mg h/L (*n* = 8), and of omadacycline orally administered at 300 mg q12 h was 20.4 ± 3.2 mg h/L (*n* = 6), which was derived from the simulation with the non-parametric superposition method.

**FIGURE 4:**
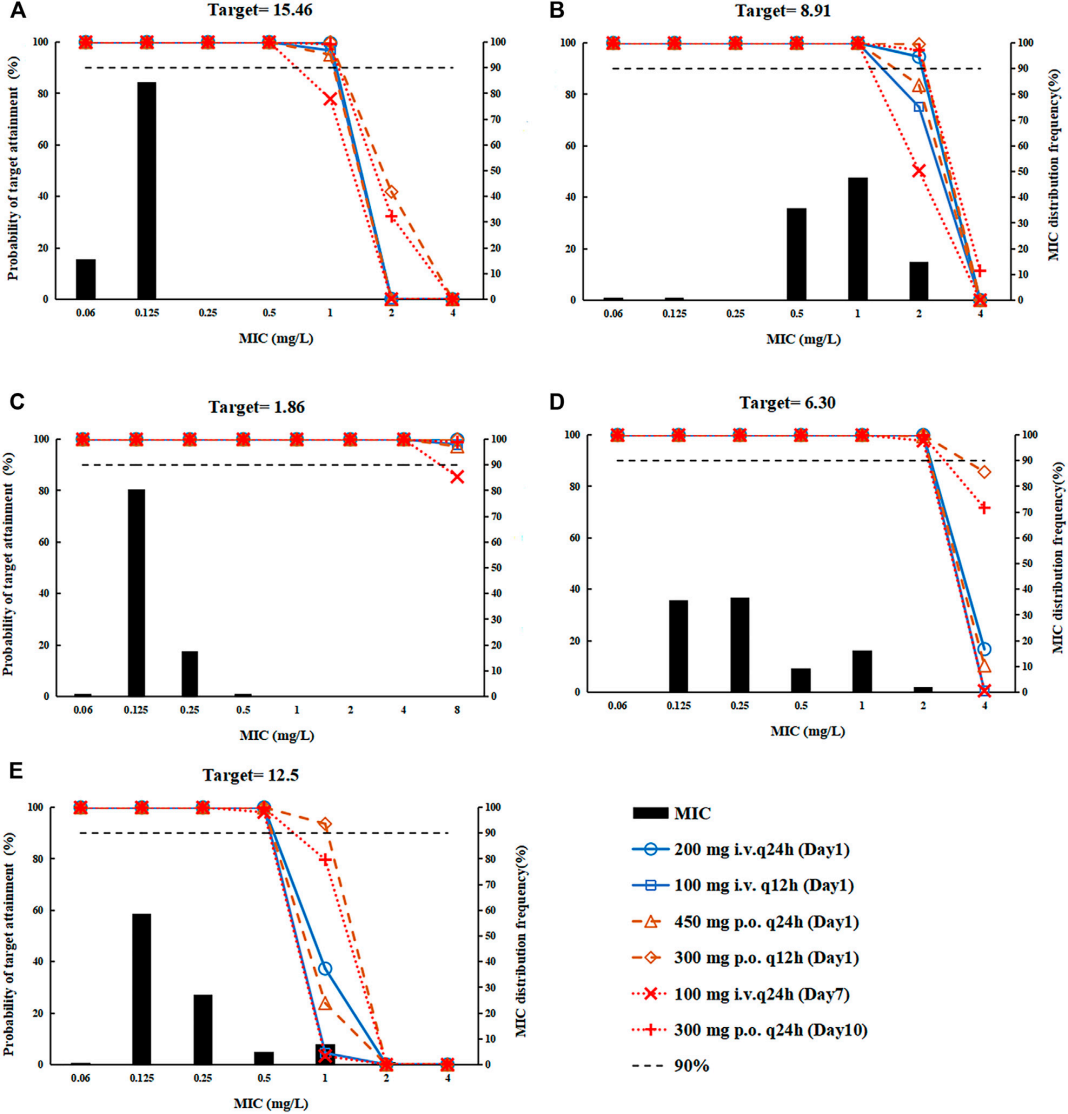
The probability of target attainment for the dosing regimens, covering *Streptococcus pneumonia*, *Haemophilus influenzae*, and *Staphylococcus aureus*. **(A)** *Streptococcus pneumoniae*; **(B)** *Haemophilus influenzae*; **(C)** methicillin-sensitive *Staphylococcus aureus* (MSSA); **(D)** methicillin-resistant *Staphylococcus aureus* (MRSA); and **(E)** *Staphylococcus aureus*.

**PK/PD Analysis** The PTA of the omadacycline FDA-approved dose regimen in the Chinese population was shown in Figure 4, and the CFR was shown in Supplementary Table S2. All dosing regimens could cover the main pathogens of the indications ABSSSI and CABP: *Staphylococcus aureus* and *Streptococcus pneumoniae*. For *Streptococcus pneumoniae*, all dosing regimens could cover a PTA >90% for MIC values of ≤0.5 mg/L (Figure 4A). For *Haemophilus influenzae*, a loading dose of 200 mg i. v q24 h or 300 mg p. o. q12 h followed by a maintenance dose of 300 mg p. o. could cover a PTA >90% and a CFR >90% for MIC values of ≤2 mg/L (Figure 4B). Although the PK/PD target value of MRSA was higher than that of MSSA, all dosing regimens could cover a PTA >90% for MIC values of≤2 mg/L (Figure 4D). For *Staphylococcus aureus* targeted ABSSSI, all dosing regimens could cover a PTA >90% for MIC values of≤ 0.5 mg/L (Figure 4E).

**Safety** A total of 33 (66.0%) subjects who received omadacycline reported treatment-emergent adverse events (TEAE), and 8 (61.5%) subjects who received placebo reported TEAEs, but no serious adverse events (SAE) occurred. One subject who received a single dose of 100 mg omadacycline intravenous infusion reported a TEAE of CTCAE grade 3: blood creatine phosphokinase increased to 5.7 times the upper limit of normal, and the subject recovered without intervention. The investigator judged that this AE was related to physical exercise and was not related to the study drug. All the other TEAEs were of grade 1.

Drug-related AEs with a frequency of ≥10% included: alanine aminotransferase (ALT) increase in seven subjects (14.0%), vomiting in six subjects (12.0%), and white blood cell count decrease in six subjects (12.0%). All drug-related AEs were resolved over time. 9 subjects (18.0%) in overall omadacycline group showed an elevation of triglycerides, the incidence rate was similar to that of the placebo group (3 cases, 23.1%). Those events were assessed as unrelated to the study drug by the investigator.

## Discussion

This study fully illustrated the PK characteristics and safety of omadacycline in the Chinese population and provided the rational of using the current dosing regimen approved by US FDA in Chinese population through PK/PD analysis.

In this study, C_max_ (1.83 *vs* 1.8–1.9 mg/L), AUC_0-inf_ (11.9 *vs* 9.76–11.3 mg h/L), and t_1/2_ (19.1 *vs* 16.3–17.1 h) of omadacycline intravenously infused at 100 mg in Chinese healthy subjects were generally comparable with those in western subjects (Sun et al., 2016; Berg et al., 2018; Kovacs et al., 2020b). The C_max_ (1.05 *vs* 0.5 mg/L) and AUC_0-inf_ (20.4 *vs* 10.3 mg h/L) of omadacycline orally administered at 300 mg were higher than those in western subjects (Sun et al., 2016), which may be attributed to the high bioavailability of Chinese subjects (42.0–62.3 *vs* 34.5%), inter-individual variability, and differences in study centers (the absorption of omadacycline that was susceptible to diet would also increase variability between individuals and studies). Moreover, Chinese subjects of the oral groups (150–450 mg) showed saturation of absorption, and T_max_ increased with the increase of the dose (T_max_ was 1.75, 3, and 4 h), while the T_max_ of omadacycline orally administered at 300–600 mg in western subjects was 2.5 h (Bundrant et al., 2018).

After intravenous infusion of omadacycline, there was a rapid distribution of α-phase (t_1/2,α_ = 0.1 h), and the β-phase of intravenous infusion was similar to α-phase of oral administration (intravenous t_1/2,β_ = 1.9 h *vs* oral administration t_1/2,α_ = 2.1 h). The γ-phase of intravenous infusion was similar to the β-phase of oral administration (intravenous t_1/2,γ_ = 18.7 h *vs* oral t_1/2,β_ = 22.6 h), so the intravenous infusion of omadacycline conformed to the pharmacokinetic characteristics of the three-compartment model and oral administration of omadacycline to the pharmacokinetic characteristics of the two-compartment model. The PK parameters of the three-compartment model established in this study based on the intravenous infusion of omadacycline in the Chinese population were similar to population pharmacokinetics (PPK) of the western population (Sun et al., 2016). The PPK study showed that the clearance of females was lower than that of males, and this study also showed that females’ exposure was slightly higher. The AUC ratios of females/males in each dose group ranged from 1.1 to 1.5 (Supplementary Figure S3 and Table S3). In this study, the double-peak absorption of omadacycline was found in the Chinese population. It was guessed that the alkaline omadacycline first mainly existed as an ionized form in the strongly acidic stomach with slow absorption, and then the remaining amount of the drug entered the weak alkaline intestinal tract with fast absorption as molecule form mainly, which may provide an explanation for the obvious double-peak absorption in 450 mg group representing, and non-separated double peak in the 150 mg group and 300 mg group representing two absorption phases (slow absorption followed by fast absorption). Previous literature reported that the oral absorption of omadacycline manifested as delayed absorption (Sun et al., 2016; Bundrant et al., 2018; Lakota et al., 2020).

The comparable exposure of 100 mg omadacycline i.v. and 300 mg omadacycline p.o. (Sun et al., 2016) supported the dosing regimen transferred from intravenous administration to oral administration, which was convenient for discharged patients to take medicine. In the Chinese population, the AUC_0-24_ of the four loading doses (200 mg i.v. q24h, 100 mg i.v. q12 h, 450 mg p. o. q24 h, or 300 mg p. o. q12 h) recommended on the FDA label were 15.3, 13.2, 14.2 and 20.4 mg h/L, respectively, comparable with that of maintenance doses (12.1 and 19.4 mg h/L for 100 mg i.v. q24 h or 300 mg p. o. q24 h), supporting the loading dose.

MRSA omadacycline MIC90 values from two different studies on isolates from China vary with one study reporting an MIC90 value of 1 mg/L (Sun et al., 2016) and a second study reporting an MIC90 value of 0.25 mg/L (Xiao et al., 2020). The former study also included assessment of MRSA isolates collected from the west and reported an MIC90 value of 0.25 mg/L (Pfaller et al., 2020) similar to Xiao et al., 2020 data for China. Regardless, the dosing regimen present on the FDA label provides >90% PTA for isolates covering both of these MIC90 values. This dosing regimen could also treat *Streptococcus pneumoniae* (MIC_90_ = 0.12 mg/L) and MSSA (MIC_90_ = 0.25 mg/L) infections with low MIC_90_, and support the indications of CABP. For *Haemophilus influenzae*, MIC_90_ in China (MIC_90_ = 2 mg/L) was higher than that in the western (1 mg/L) (Pfaller et al., 2020), whereas the dosing regimen of a loading-dose of 200 mg i.v. or 300 mg p. o. followed by a maintenance dose of 300 mg p. o. could achieve PTA>90% in the Chinese population, and all dosing regimens could reach CFR>90%, supporting the treatment with omadacycline for CABP infected by *Haemophilus influenzae* in the Chinese population. Phase III clinical trial in the western also reported the good efficacy of omadacycline in the treatment of patients with pneumonia infected by *Haemophilus influenzae* (MIC_90_ = 2 mg/L) (Pfaller et al., 2020). For ABSSSI patients, all administration methods could reach the target value of 96% early clinical response.

This study has some limitations. T_1/2_ of omadacycline in intravenous 150 mg group and oral 300 and 450 mg group ranged from 19.7 to 20.7 h. However, the sampling period was 96 h which did not cover 5 times of T_1/2_. Although AUC/MIC targets were based on specific infection types, the MIC data used for PTA and CFR calculations were not stratified by infection types. Since population PK analyses were not performed in this article, covariates and incorporate inter-individual variation were not assessed. Simulated values instead of measured values were adopted for omadacycline 200 mg i.v. q24 h, 100 mg i.v. q12 h and 300 mg p. o. q12 h but it was believed that the linearity between exposure and dose in the 50–150 mg group supported the extrapolation of the simulation results. Furthermore, it was found that the oral exposure to omadacycline in China was significantly higher than that in western subjects (the AUC ratio was 1.98), having no noticeable influence on the safety and tolerance of subjects. The mechanism is still unclear and accidental factors cannot be ruled out due to the small sample size of this study, so further studies would be conducted.

In this study, seven subjects (13.5%) had transient elevated ALT after administration of omadacycline, with aspartate aminotransferase (AST) and bilirubin in the normal range. The same phenomenon was observed in healthy subjects in the literature (Bundrant et al., 2018; Overcash et al., 2019). However, it is reported elevated ALT and AST in patients (Abrahamian et al., 2019; O'Riordan et al., 2019; Stets et al., 2019). AEs regarding infusion site-related reactions have been reported in western studies of healthy subjects and patients (Abrahamian et al., 2019; O'Riordan et al., 2019; Stets et al., 2019), but in this study, there were no findings on any AEs with infusion site reaction.

## Conclusion

This study demonstrated that omadacycline had a good safety profile in Chinese healthy subjects. Oral administration manifested as double-peak absorption, and its pharmacokinetic characteristics were similar to those of western populations. PK/PD analysis demonstrated the rationality of the loading dose (200 mg i.v. q24 h, 100 mg i.v. q12 h, 450 mg p. o. q24 h × 2 days, or 300 mg p. o. q12 h) and maintenance dose (100 mg i.v. q24 h or 300 mg p. o. q24 h) of omadacycline for the treatment of Chinese ABSSSI and CABP patients.

## Data Availability

The raw data supporting the conclusion of this article will be made available by the authors, without undue reservation.

## References

[B1] Abrahamian F. M. Sakoulas G. Tzanis E. Manley A. Steenbergen J. Das A. F. (2019). Omadacycline for Acute Bacterial Skin and Skin Structure Infections. Clin. Infect. Dis. 69 (Suppl. 1), S23–S32. 10.1093/cid/ciz396 31367742 PMC6669297

[B2] Berg J. K. Tzanis E. Garrity-Ryan L. Bai S. Chitra S. Manley A. (2018). Pharmacokinetics and Safety of Omadacycline in Subjects with Impaired Renal Function. Antimicrob. Agents Chemother. 62 (2). 10.1128/AAC.02057-17 PMC5786750 29158281

[B3] Bundrant L. A. Tzanis E. Garrity-Ryan L. Bai S. Chitra S. Manley A. (2018). Safety and Pharmacokinetics of the Aminomethylcycline Antibiotic Omadacycline Administered to Healthy Subjects in Oral Multiple-Dose Regimens. Antimicrob. Agents Chemother. 62 (2). 10.1128/AAC.01487-17 PMC5786815 29180524

[B4] Carvalhaes C. G. Huband M. D. Reinhart H. H. Flamm R. K. Sader H. S. (2019). Antimicrobial Activity of Omadacycline Tested against Clinical Bacterial Isolates from Hospitals in Mainland China, Hong Kong, and Taiwan: Results from the SENTRY Antimicrobial Surveillance Program (2013 to 2016). Antimicrob. Agents Chemother. 63 (3). 10.1128/AAC.02262-18 PMC6395890 30617092

[B5] Dong D. Zheng Y. Chen Q. Guo Y. Yang Y. Wu S. (2020). *In Vitro* activity of Omadacycline against Pathogens Isolated from Mainland China during 2017-2018. Eur. J. Clin. Microbiol. Infect. Dis. 39 (8), 1559–1572. 10.1007/s10096-020-03877-w 32356026

[B6] Draper M. P. Weir S. Macone A. Donatelli J. Trieber C. A. Tanaka S. K. (2014). Mechanism of Action of the Novel Aminomethylcycline Antibiotic Omadacycline. Antimicrob. Agents Chemother. 58 (3), 1279–1283. 10.1128/AAC.01066-13 24041885 PMC3957880

[B7] Gotfried M. H. Horn K. Garrity-Ryan L. Villano S. Tzanis E. Chitra S. (2017). Comparison of Omadacycline and Tigecycline Pharmacokinetics in the Plasma, Epithelial Lining Fluid, and Alveolar Cells of Healthy Adult Subjects. Antimicrob. Agents Chemother. 61 (9). 10.1128/AAC.01135-17 PMC5571291 28696233

[B8] Huang Z. W. Yu J. C. Wang J. J. Chen Y. C. Wu J. F. Chen Y. J. (2021). Pharmacokinetics and Safety of Single-Dose Amphotericin B Colloidal Dispersion in Healthy Chinese Subjects and Population Pharmacokinetic/Pharmacodynamic Analysis to Inform Clinical Efficacy in Invasive Infections Caused by Candida Albicans. Clin. Ther. 43 (11), 1921–1933. e7. 10.1016/j.clinthera.2021.09.012 34686365

[B9] Karlowsky J. A. Steenbergen J. Zhanel G. G. (2019). Microbiology and Preclinical Review of Omadacycline. Clin. Infect. Dis. 69 (Suppl. 1), S6–S15. 10.1093/cid/ciz395 31367743 PMC6669291

[B10] Kovacs S. J. Ting L. Praestgaard J. Sunkara G. Sun H. Stein D. S. (2020a). An Open-Label Study of the Impact of Hepatic Impairment on the Pharmacokinetics and Safety of Single Oral and Intravenous Doses of Omadacycline. Antimicrob. Agents Chemother. 64 (11). 10.1128/AAC.01650-20 PMC7577144 32839218

[B11] Kovacs S. J. Ting L. Praestgaard J. Sunkara G. Sun H. Stein D. S. (2020b). Correction for Kovacs et alAn Open-Label Study of the Impact of Hepatic Impairment on the Pharmacokinetics and Safety of Single Oral and Intravenous Doses of Omadacycline. Antimicrob. Agents Chemother. 65 (1). 10.1128/AAC.02426-20 PMC7927805 33328319

[B12] Lakota E. A. Van Wart S. A. Trang M. Tzanis E. Bhavnani S. M. Safir M. C. (2020). Population Pharmacokinetic Analyses for Omadacycline Using Phase 1 and 3 Data. Antimicrob. Agents Chemother. 64 (7). 10.1128/AAC.02263-19 PMC7318031 32340986

[B13] Lepak A. J. Zhao M. Marchillo K. Vanhecker J. Andes D. R. (2020). *In Vivo* Pharmacodynamic Evaluation of Omadacycline against *Staphylococcus aureus* in the Neutropenic Mouse Pneumonia Model. Antimicrob. Agents Chemother. 64 (2). 10.1128/AAC.02058-19 PMC6985749 31712210

[B14] Lepak A. J. Zhao M. Marchillo K. Vanhecker J. Andes D. R. (2017). *In Vivo* Pharmacodynamic Evaluation of Omadacycline (PTK 0796) against Streptococcus Pneumoniae in the Murine Pneumonia Model. Antimicrob. Agents Chemother. 61 (5). 10.1128/AAC.02368-16 PMC5404567 28193651

[B15] Lin C. C. Arkenau H. T. Lu S. Sachdev J. de Castro C. J. Mita M. (2019). A Phase 1, Open-Label, Dose-Escalation Trial of Oral TSR-011 in Patients with Advanced Solid Tumours and Lymphomas. Br. J. Cancer 121 (2), 131–138. 10.1038/s41416-019-0503-9 31217479 PMC6738096

[B16] Lin W. Flarakos J. Du Y. Hu W. He H. Mangold J. (2017). Pharmacokinetics, Distribution, Metabolism, and Excretion of Omadacycline Following a Single Intravenous or Oral Dose of 14C-Omadacycline in Rats. Antimicrob. Agents Chemother. 61 (1). 10.1128/AAC.01784-16 PMC5192155 27821446

[B17] Noel A. R. Attwood M. Bowker K. E. Macgowan A. P. (2021). *In Vitro* pharmacodynamics of Omadacycline against *Escherichia coli* and Acinetobacter Baumannii. J. Antimicrob. Chemother. 76 (3), 667–670. 10.1093/jac/dkaa508 33294925

[B18] O'Riordan W. Green S. Overcash J. S. Puljiz I. Metallidis S. Gardovskis J. (2019). Omadacycline for Acute Bacterial Skin and Skin-Structure Infections. N. Engl. J. Med. 380 (6), 528–538. 10.1056/NEJMoa1800170 30726689

[B19] Overcash J. S. Bhiwandi P. Garrity-Ryan L. Steenbergen J. Bai S. Chitra S. (2019). Pharmacokinetics, Safety, and Clinical Outcomes of Omadacycline in Women with Cystitis: Results from a Phase 1b Study. Antimicrob. Agents Chemother. 63 (5). 10.1128/AAC.02083-18 PMC6496050 30858208

[B20] Pfaller M. A. Huband M. D. Shortridge D. Flamm R. K. (2020). Surveillance of Omadacycline Activity Tested against Clinical Isolates from the United States and Europe: Report from the SENTRY Antimicrobial Surveillance Program, 2016 to 2018. Antimicrob. Agents Chemother. 64 (5). 10.1128/AAC.02488-19 PMC7179604 32071045

[B21] Stets R. Popescu M. Gonong J. R. Mitha I. Nseir W. Madej A. (2019). Omadacycline for Community-Acquired Bacterial Pneumonia. N. Engl. J. Med. 380 (6), 517–527. 10.1056/NEJMoa1800201 30726692

[B22] Sun H. Ting L. Machineni S. Praestgaard J. Kuemmell A. Stein D. S. (2016). Randomized, Open-Label Study of the Pharmacokinetics and Safety of Oral and Intravenous Administration of Omadacycline to Healthy Subjects. Antimicrob. Agents Chemother. 60 (12), 7431–7435. 10.1128/AAC.01393-16 27736760 PMC5119026

[B23] Tzanis E. Manley A. Villano S. Tanaka S. K. Bai S. Loh E. (2017). Effect of Food on the Bioavailability of Omadacycline in Healthy Participants. J. Clin. Pharmacol. 57 (3), 321–327. 10.1002/jcph.814 27539539 PMC5324643

[B24] U. S. Department of Health and Human Services (2010). Availableat: https://ctep.cancer.gov/protocolDevelopment/electronic_applications/ctc.htm#ctc_40 (Accessed June 14, 2010). 10.3109/15360288.2015.1037530 26095483

[B25] Xiao M. Huang J. J. Zhang G. Yang W. H. Kong F. Kudinha T. (2020). Antimicrobial Activity of Omadacycline *In Vitro* against Bacteria Isolated from 2014 to 2017 in China, a Multi-center Study. BMC Microbiol. 20 (1), 350. 10.1186/s12866-020-02019-8 33198626 PMC7667747

