# Supplementary material for: Pharmacokinetics, Safety and Pharmacokinetics/Pharmacodynamics Analysis of Omadacycline in Chinese Healthy Subjects
Source: Front Pharmacol. 2022 Apr 21;13:869237. doi: 10.3389/fphar.2022.869237 (PMC9068897; doi:10.3389/fphar.2022.869237)
Supplement: Supplementary file 1 [file DataSheet1.docx]

**Supplementary**

**Results**

**Supplementary Table 1** Demographic characteristics of intravenous and oral administration group of Omadacycline

|  | Intravenous administration | | | | Intravenous placebo | | Oral administration | | | | Oral placebo | |
| --- | --- | --- | --- | --- | --- | --- | --- | --- | --- | --- | --- | --- |
|  | 50 mg single dose | 100 mg single dose | 100 mg  multiple  doses | 150 mg  single dose | single dose | multiple  doses | 150 mg  single dose | 300 mg  single dose | 300 mg multiple  doses | 450 mg  single dose | single dose | multiple  doses |
| *n* | 8 | 8 | | 8 | 6 | 2 | 8 | 8 | 8 | 8 | 6 | 2 |
| Male (%) | 50 | 50 | | 50 | 50 | 50 | 50 | 37.5 | 37.5 | 50 | 66.7 | 100 |
| Age (year) | 24.1 ± 3.7 | 28.0 ± 5.4 | | 26.9 ± 5.2 | 29.5 ± 4.0 | 32.5 ± 5.0 | 28.6 ± 7.0 | 30.5 ± 2.5 | 30.3 ± 2.3 | 28.3 ± 4.0 | 26.7 ± 2.9 | 33.0 ± 8.5 |
| Height (cm) | 165 ± 8.2 | 163 ± 8.2 | | 162 ± 4.6 | 164 ± 11 | 162 ± 18 | 164 ± 11 | 164 ± 8.3 | 165 ± 7.2 | 163 ± 7.3 | 168 ± 5.5 | 174 ± 2.4 |
| Weight (kg) | 59.3 ± 8.9 | 60.3 ± 7.5 | | 60.5 ± 8.0 | 60.7 ± 6.9 | 60.8 ± 13 | 63.6 ± 9.1 | 60.8 ± 9.0 | 61.7 ± 7.2 | 60.6 ± 9.7 | 61.4 ± 8.0 | 72.7 ± 3.7 |
| Body mass index (kg/m^2^) | 21.7 ± 2.3 | 22.7 ± 1.8 | | 23.1 ± 2.6 | 22.6 ± 1.1 | 23.0 ± 0.35 | 23.1 ± 1.7 | 22.4 ± 2.2 | 22.6 ± 2.2 | 22.8 ± 1.8 | 21.8 ± 1.8 | 23.9 ± 0.57 |
| Asian (%) | 100 | 100 | | 100 | 100 | 100 | 100 | 100 | 100 | 100 | 100 | 100 |
| Albumin (g/L) | 46.5 ± 2.3 | 46.9 ± 2.4 | 44.1 ± 0.8 | 48.0 ± 2.7 | 47.2 ± 2.6 | 44.5 ± 2.1 | 46.9 ± 2.4 | 44.5 ± 1.7 | 47.4 ± 3.0 | 45.9 ± 3.0 | 46.2 ± 1.6 | 47.0 ± 1.4 |
| Creatinine  (μmol/L) | 65.5 ± 12 | 62.3 ± 10 | 61.9 ± 8.8 | 60.4 ± 17 | 65.8 ± 16 | 70.0 ± 24 | 65.3 ± 18 | 62.4 ± 9.6 | 59.6 ± 17 | 64.0 ± 9.9 | 69.8 ± 23 | 89.5 ± 19 |
| ALT (U/L) | 13.5 ± 3.2 | 13.3 ± 4.0 | 14.1 ± 7.2 | 11.0 ± 5.0 | 16.8 ± 8.8 | 17.0 ± 0.0 | 13.9 ± 7.6 | 12.3 ± 4.0 | 15.5 ± 5.6 | 11.1 ± 3.6 | 13.2 ± 5.6 | 27.5 ± 12 |
| AST (U/L) | 16.6 ± 1.9 | 17.4 ± 2.8 | 18.1 ± 5.6 | 17.8 ± 2.6 | 18.3 ± 1.6 | 17.5 ± 2.1 | 16.8 ± 3.8 | 15.4 ± 1.5 | 17.3 ± 2.6 | 16.3 ± 1.8 | 18.0 ± 3.0 | 21.0 ± 5.7 |

100 mg single dose group and 100 mg multiple dose group were the same subjects. 6 subjects were the same in 300 mg single dose group and 300 mg multiple dose group; ALT: alanine aminotransferase; AST: aspartate aminotransferase.

**Supplementary Table 2** The cumulative fraction of response (%) of Omadacycline dosing regimen in the Chinese population

| Dosing regimen | CABP | | | |  | ABSSSI |
| --- | --- | --- | --- | --- | --- | --- |
|  | *Streptococcus pneumoniae* | *Haemophilus influenzae* | MSSA | MRSA |  | *Staphylococcus aureus* |
| 200 mg i.v. q24h (Day1) | 100 | 99 | 100 | 100 |  | 94 |
| 100 mg i.v. q12h (Day1) | 100 | 96 | 100 | 100 |  | 91 |
| 450 mg p.o. q24h (Day1) | 100 | 98 | 100 | 100 |  | 93 |
| 300 mg p.o. q12h (Day1) | 100 | 100 | 100 | 100 |  | 98 |
| 100 mg i.v. q24h (Day7) | 100 | 93 | 100 | 100 |  | 91 |
| 300 mg p.o. q24h (Day10) | 100 | 100 | 100 | 100 |  | 97 |

ABSSSI acute bacterial skin and skin structure infections; CABP community-acquired bacterial pneumonia; MRSA methicillin-resistant *Staphylococcus aureus*; MSSA methicillin-sensitive *Staphylococcus aureus*

**Supplementary Table 3** Differences between male and female subjects on the exposure of Omadacycline following intravenous and oral administrations (Mean and CV%)

|  | Dose group | Gender | *n* | AUC_0-24_ (μg•h/mL) | AUC_0-inf_ (μg•h/mL) | AUC_0-tau_ (μg•h/mL) |
| --- | --- | --- | --- | --- | --- | --- |
| Intravenous administration | 50 mg | male | 4 | 3.47 (6.5) | 5.37 (7.6) | / |
|  |  | female | 4 | 4.16 (0.8) | 6.03 (2.9) | / |
|  | 100 mg (single dose) | male | 4 | 7.06 (6.9) | 10.9 (7.2) | / |
|  |  | female | 4 | 8.53 (4.9) | 13.0 (11.0) | / |
|  | 100 mg (steady state) | male | 4 | / | / | 10.4 (5.7) |
|  |  | female | 4 | / | / | 13.7 (9.2) |
|  | 150 mg | male | 4 | 9.92 (8.6) | 12.9 (11.6) | / |
|  |  | female | 4 | 16.4 (10) | 19.7 (14.7) | / |
| Oral administration | 150 mg | male | 4 | 6.02 (10.2) | 9.72 (9.1) | / |
|  |  | female | 4 | 7.57 (19.7) | 11.6 (19.7) | / |
|  | 300 mg (single dose) | male | 2 | 9.83 (11.2) | 15.5 (10.0) | / |
|  |  | female | 4 | 13.7 (4.4) | 22.9 (3.1) | / |
|  | 300 mg (steady state) | male | 3 | / | / | 14.9 (6.8) |
|  |  | female | 5 | / | / | 22.1 (7.2) |
|  | 450 mg | male | 3 | 12.9 (7.2) | 15.4 (16.7) | / |
|  |  | female | 3 | 21.3 (6.4) | 24.1 (14.8) | / |

AUC_0-24_ area under the concentration-time curve from 0 to 24; AUC_0-inf_ the AUC from 0 to infinity; AUC_tau_ the steady-state dosing interval AUC


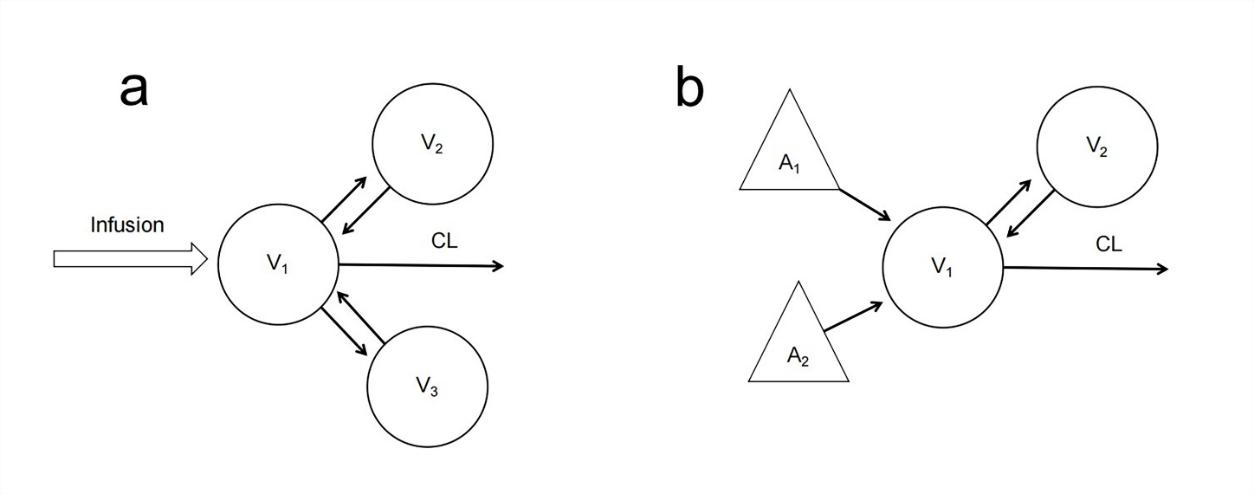


**Supplementary Figure 1.** The Structure diagram of the compartmental model of intravenous and oral administration of Omadacycline. a: The intravenous administration of Omadacycline conforms to the three-compartment model; b: the oral administration of Omadacycline conforms to the two-compartment model with two absorption compartments; A_1_ and A_2_ absorption compartment; CL clearance; V_1_ central compartment; V_2_ and V_3_ peripheral compartments; arrows indicate drug transport pathways


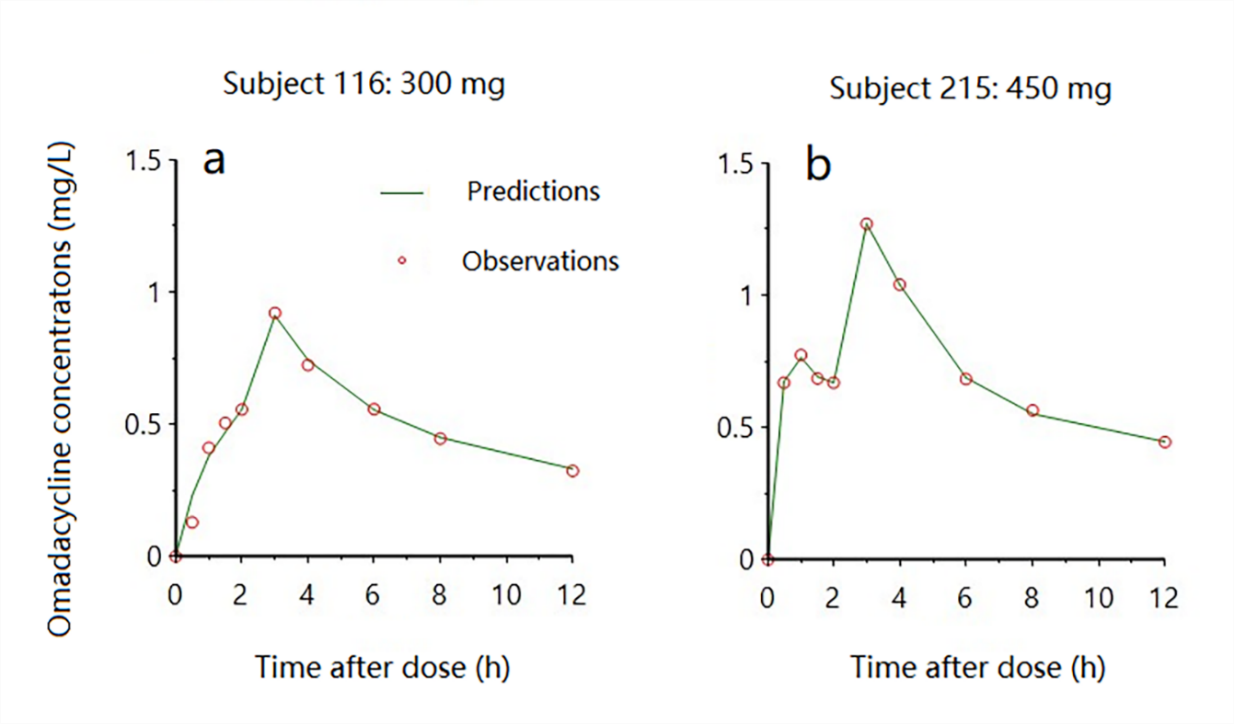


**Supplementary Figure 2.** Fitting diagram of compartmental model of oral Omadacycline in some individuals. a: subject 116 in 300 mg group; b: subject 215 in 450 mg group). The 450 mg group showed a double-peak absorption curve, and the 300 mg group showed non-separated double-peak with two absorption phases, slow absorption first and then quick absorption.


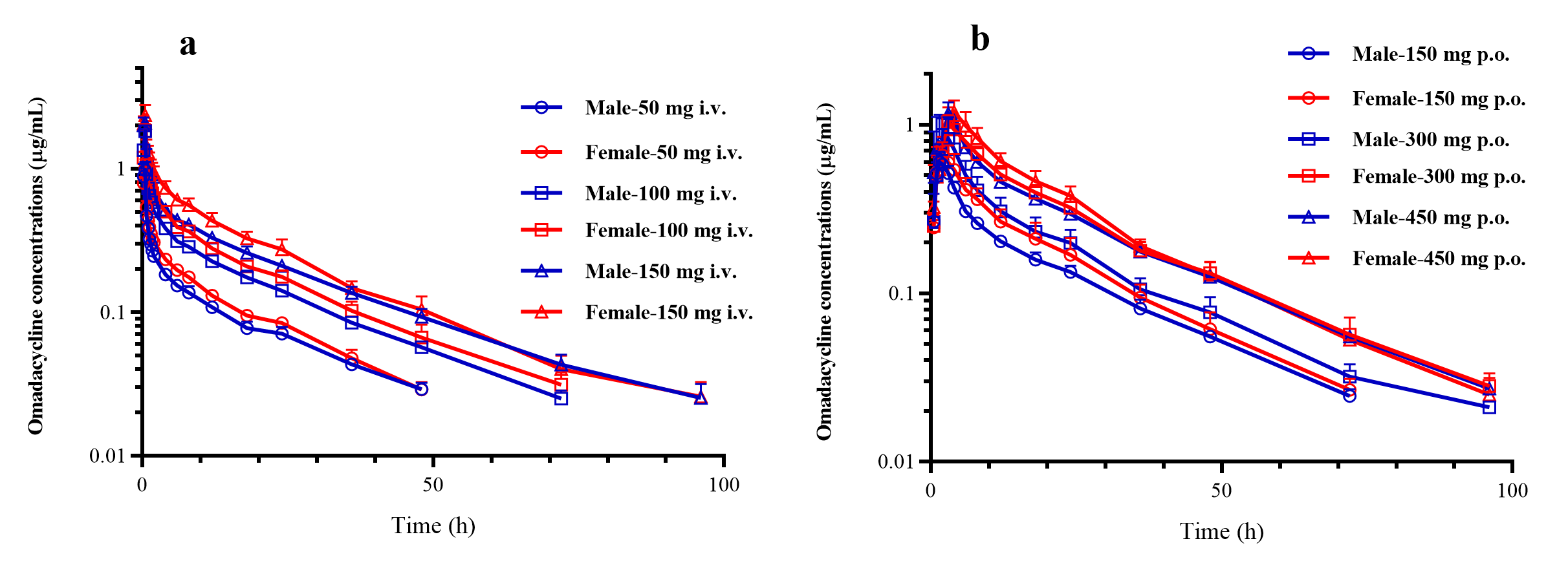


**Supplementary Figure 3.** Differences between male and female subjects on the plasma concentration-time curve of Omadacycline. a: Omadacycline following intravenous administrations; b: Omadacycline following oral administrations
